# Helping Eve Overcome ADAM: G-Quadruplexes in the ADAM-15 Promoter as New Molecular Targets for Breast Cancer Therapeutics

**DOI:** 10.3390/molecules181215019

**Published:** 2013-12-05

**Authors:** Robert V. Brown, Vanessa C. Gaerig, Taesha Simmons, Tracy A. Brooks

**Affiliations:** 1 College of Pharmacy, The University of Arizona, Tucson, AZ 85721, USA; E-Mails: rbrown1@email.arizona.edu (R.V.B.); vgaerig@email.arizona.edu (V.C.G.); 2 Department of Pharmacology, School of Pharmacy, The University of Mississippi, University, MS 38677, USA; E-Mail: taeshasimmons@yahoo.com

**Keywords:** G-quadruplex, ADAM-15, breast cancer

## Abstract

ADAM-15, with known zymogen, secretase, and disintegrin activities, is a catalytically active member of the ADAM family normally expressed in early embryonic development and aberrantly expressed in various cancers, including breast, prostate and lung. ADAM-15 promotes extracellular shedding of E-cadherin, a soluble ligand for the HER2/neu receptor, leading to activation, increased motility, and proliferation. Targeted downregulation of both ADAM-15 and HER2/neu function synergistically kills breast cancer cells, but to date there are no therapeutic options for decreasing ADAM-15 function or expression. In this vein, we have examined a unique string of guanine-rich DNA within the critical core promoter of ADAM-15. This region of DNA consists of seven contiguous runs of three or more consecutive guanines, which, under superhelical stress, can relax from duplex DNA to form an intrastrand secondary G-quadruplex (G4) structure. Using biophysical and biological techniques, we have examined the G4 formation within the entire and various truncated regions of the ADAM-15 promoter, and demonstrate strong intrastrand G4 formation serving to function as a biological silencer element. Characterization of the predominant G4 species formed within the ADAM-15 promoter will allow for specific drug targeting and stabilization, and the further development of novel, targeted therapeutics.

## 1. Introduction

The ‘A Disintegrin And Metalloproteinase’ (ADAM, alluding to their relationship to snake venom and involvement with sperm and fertility) protein family is over 20 members strong, approximately half of which have functional enzymatic activity [1,2]. The biological processes in which the ADAM proteins are involved are varied, and in addition to the aforementioned venom and sperm involvement also include nervous system cell fate, cellular migration, muscle development, ectodomain shedding, and the immune and inflammatory response. Pathologically, the ADAMs have a protective function in Alzheimer’s disease via processing of the amyloid precursor protein, but their dysregulation is involved in many oncogenic processes. Many family members are upregulated in various cancers, notably including ADAMs-10, -15, and -17, where they enhance malignancy via stimulated cellular proliferation, angiogenic processes, and invasion and metastases [1,3,4,5].

ADAM-15 is one of the catalytically active family members, and the only one with a true disintegrin-domain [6,7]. In normal physiology, its expression is widespread throughout somatic tissues. Homozygous null mice demonstrate no overt phenotype, although decreased angiogenesis was noticed with models of retinopathy and melanoma, and the onset of osteoarthritis was earlier [8,9]. Increased ADAM-15 mRNA and protein has been noted in lung, prostate, and breast cancer, and overexpression in breast cancer is coincidental with Her2/neu expression and a more aggressive and invasive phenotype [10,11,12]. Possible mechanisms for the promotion of cancer progression by ADAM-15 include disrupted cell adhesion, sheddase activity promoting autocrine and paracrine signaling, the breakdown of extracellular matrix and basement membranes, and a role in neovascularization and angiogenesis [13]. *In vivo* synergy between a HER2/neu-targeted agent and ADAM-15 knockdown with siRNA was demonstrated [14], highlighting the promise of targeting a single pathway at multiple points early in the signaling cascade. With the clear involvement of ADAM-15 in numerous aspects of tumorigenesis, invasion and metastasis, pharmaceutical targeting is attractive. However, to date there is no molecular target available to focused ADAM-15 modulation or downregulation.

We have identified a unique region of guanine-rich DNA in the critical core promoter of ADAM-15. This region DNA consists of seven contiguous runs of three or more consecutive guanines within a region 40 nucleotides in length comprising the core promoter, known to have a multiple binding sites for the transcriptional factor Sp1 and to be involved in regulating gene expression [15]. Negative superhelicity induced by transcription results in polyguanine/polycytosine-rich DNA region opening up to form unique secondary structures: G-quadruplexes (G4s). G4 structures are formed when two or more tetrads stack, each tetrad comprised of four guanines bonded by Hoogsteen hydrogen bonds and stabilized with monovalent cations. Putative G4 forming regions have at least four runs of three or more consecutive guanines separated by varying nucleotides that comprise the loop structures. Such 15^+^ base pair regions of guanine-rich DNA are preferentially clustered ±0.5 kb from the transcriptional start site [16] and within oncogenic promoters [17], including many representatives of the six hallmarks of cancer [18]. The G4s form globular structures, which allow for specific and molecular targeting by small molecule drugs. Formation in DNA modulates transcription and usually leads to a downregulation of protein expression, whereas formation in RNA has been shown to both up- and down-regulate translation [19,20,21,22]. Due to varying loop lengths and tetrad composition, drug design for G4 moieties is analogous to protein targeting a unique tertiary structure. A number of agents targeting these unique G4 DNA secondary structures are under development across the country and around the world [23]. The current works define the role of G4 formation in the ADAM-15 promoter through luciferase constructs, and elucidates the structures of the predominating isoforms through various biophysical techniques including CD, chemical footprinting, select mutations and the polymerase stop assay.

## 2. Results and Discussion

### 2.1. G-Quadruplex (G4) Formation in the ADAM-15 Promoter

**Figure 1:**
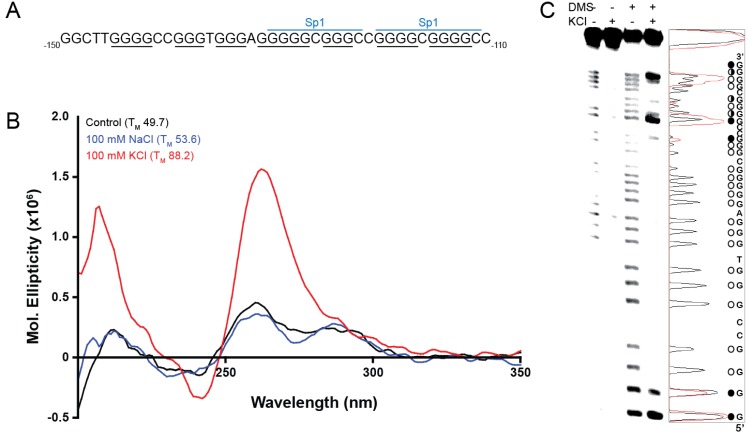
(**A**) The guanine-rich region of DNA spanning -110 to -150 bp upstream of the transcriptional start site contains seven runs of continuous G’s (underlined) and two Sp1 binding sites (spanning blue lines). This region (**B**) forms a weak, mixed species G4 in the absence of salt (black line) or 100 mM NaCl (blue line), and a stable, predominantly parallel, G4 in the presence of 100 mM KCl (red line). (**C**) DMS footprinting in the absence and presence of 140 mM KCl shows an induction of protection of a number of guanines, indicative of a mixture of G4 species. Histograms of the cleavage pattern in the absence (black line) and presence (red line) of KCl were used to determine the protection pattern indicated by the circles to the right of the figure: filled circle = hyper-cleaved, half-filled circle = partial protection indicative of protection within a subset of the equilibrating G4s, open circle = full protection indicative of incorporation in the majority of equilibrating G4 species.

Within the proximal promoter for ADAM-15 lies a guanine-rich region known to contain two binding sites for the transcriptional factor Sp1; from -112 to -145 bases from the transcriptional start site there are seven runs of at least three continuous guanines (Figure 1A). Putative G4 formation in this full-length wild-type (FL WT) sequence was examined in the absence and presence of either 100 mM NaCl or KCl by both CD spectra and thermal stability measurement. The presence of Na^+^, known to preferentially stabilize intermolecular G4 structures, minorly delineated the parallel (260–265 nm) and antiparallel (290–295 nm) spectral peaks, as compared to the control conditions, but failed to induce a strong G4 spectral signal. The T_M_ increased by approximately 4 °C, as compared to the more robust 38.5 °C increase induced by KCl, which also notably induced a predominantly parallel G4 structure (Figure 1B). DMS footprinting of the FL WT sequence in the absence and presence of 140 mM KCl highlighted a strong pattern of guanine protection concordant with G4 formation, but also demonstrated that multiple isoforms exist in equilibrium, as indicated by more than four sets of three continuous guanines undergoing protection under cationic conditions (Figure 1C).

### 2.2. G4-Mediated Transcriptional Silencing of ADAM-15

**Figure 2:**
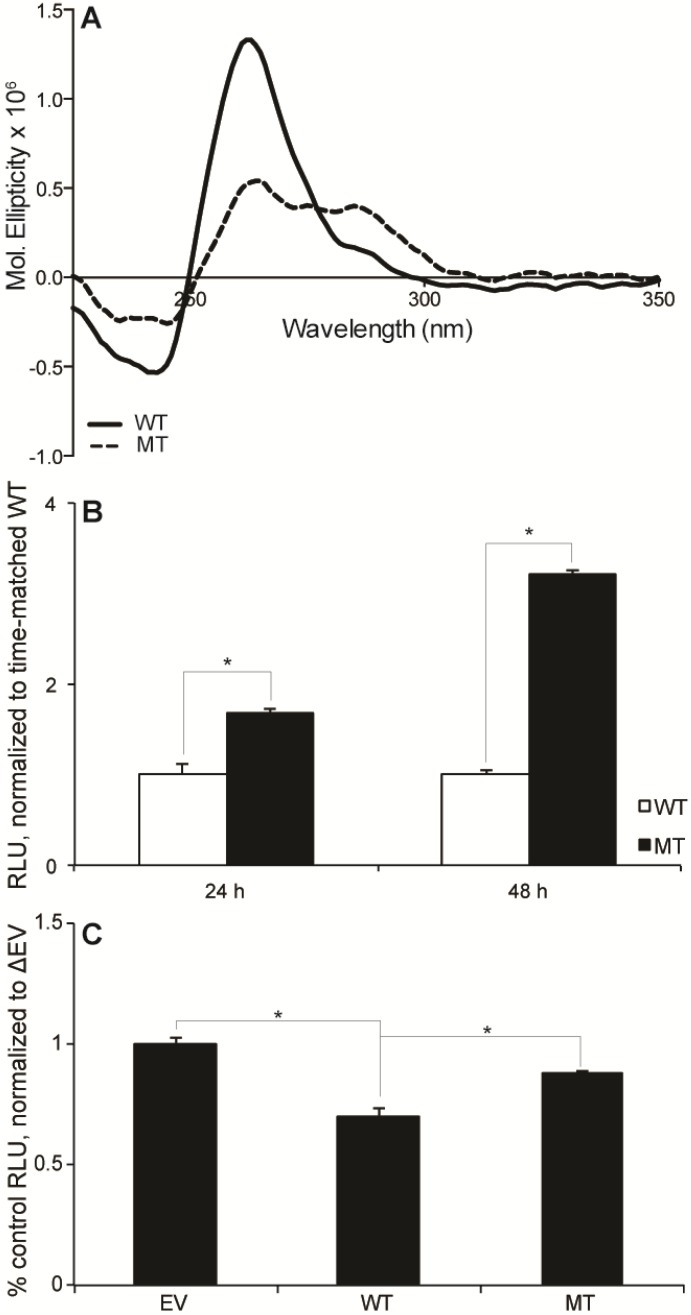
(**A**) CD of the WT and MT (a23d3g23) sequences in the presence of 100 mM KCl demonstrated the abrogation of strong G4 formation in the MT sequence, which was used for further biological experimentation. (**B**) These sequences were inserted into luciferase vectors and co-transfected into HEK-293 cells with control renilla luciferase plasmids for 24 and 48 h. Luciferase expression was normalized to renilla expression, and then to WT plasmid basal expression. (**C**) The effect of G4 stabilization on ADAM-15 promoter activity was further probed with the addition of 100 µM TMPyP4 for 48 h post-plasmid transfection. Luciferase expression was normalized as described in the Experimental section. All experiments were done in at least triplicate; * *p* < 0.05.

**Figure 3:**
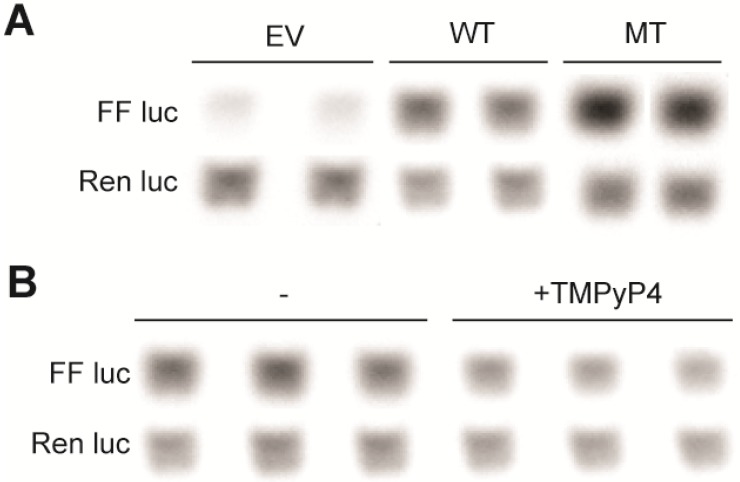
Real-time PCR of firefly and renilla luciferase from ADAM-15 vectors. (**A**) Changes in basal expression from representative duplicate experiments demonstrate the transcriptional upregulation of firefly expression from the MT, versus WT, vectors due to the lack of G4-formation. (**B**) The addition of 100 µM TMPyP4 to the WT plasmid results in a transcriptional downregulation; quantification of band density reveals a 30% decrease. For all samples, renilla luciferase expression serves as the housekeeping control.

G-to-T mutations in the first, fourth, and seventh run of continuous were used to interrupt G4 formation in the FL WT sequence. G4 formation was notably impacted under ex vivo conditions (Figure 2A). The WT and this mutant (MT) sequence were inserted into a pGL3 Basic luciferase vector, and the resultant plasmids were transiently transfected into HEK-293 cells. The inability to form a strongly inducible G4 significantly increased luciferase expression from 1.0 ± 0.1 to 1.7 ± 0.0 and from 1.0 ± 0.1 to 3.2 ± 0.1 at 24 and 48 h post-transfection, respectively (*p* < 0.05) (Figure 2B). Changes in expression are apparently due primarily to a change in transcription (rather than translation), as there is a notable increase in transcript from the MT, versus WT plasmid (Figure 3A). These findings are consistent with G4-formation mediating a silencing function, which was further tested by the addition of 100 µM TMPyP4, a pan-G4-stabilizing cationic porphyrin compound. Incubation with TMPyP4 for 48 h significantly decreased luciferase expression to 0.7 ± 0.0-fold the EV effect in the WT, but not MT plasmid (0.9 ± 0.0-fold), further supporting the biological function of G4-formation within the ADAM-15 to be a silencing event (*p* < 0.05) (Figure 2C). RT-PCR was performed for firefly and renilla luciferase in order to confirm that this silencing was at the transcriptional, rather than translational level. Indeed, the luciferase transcription significantly decreased in correlation with the functional activity to 0.7 ± 0.0-fold the EV effect in the WT plasmid (*p* < 0.01) (Figure 3B). We postulate that this increase in transcriptional output with G-to-T mutations within the G4-forming sequence is due to a lack of G4-formation rather than an alteration in the binding of Sp1 as a loss of canonical binding sites would be expected to decrease, and not increase, transcriptional activity. Our mutations in the a23d3g23 (MT) plasmid disrupted the Sp1 binding sites along with most G4 formation, and significant increase in transcription was noted. As this cannot be explained by more Sp1 binding, and the further addition of the pan-G4-stabilizing compound TMPyP4 further decreased luciferase output in the WT plasmid, our hypothesis that G4-formation is a silencing element is well supported. Future binding studies of Sp1 to a variety of DNA structures are warranted, along with illumination of all regulatory proteins of the G/C-rich region.

### 2.3. Multiple G4-Isoforms Forming within the ADAM-15 Promoter

**Figure 4:**
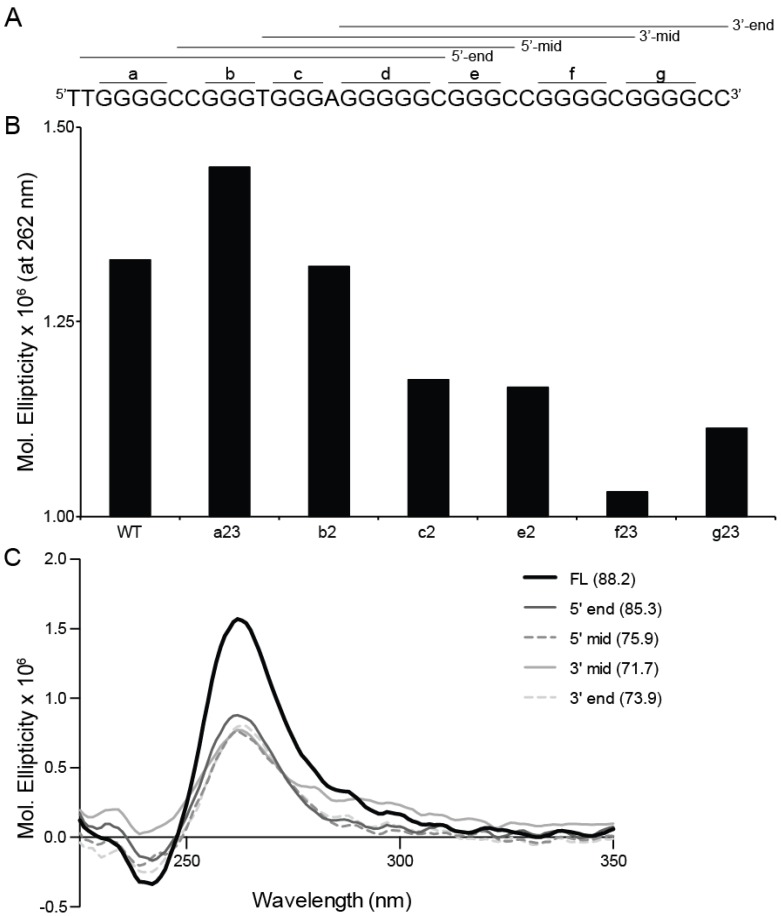
(**A**) Continuous runs of guanines in the 5'–3' ADAM-15 sequence were assigned sequential letters (a–g) and each run was mutated individually in the central 1 or 2 guanines (e.g., a23 indicates that the second and third guanine in the run of four were replaced with thymines) and used for further isoform analysis. Additionally, the sequence was truncated to the minimal sets of four contiguous runs of guanine as indicated by the 5'-end, *etc*., indicated by the lines over the sequence. CD was performed in the presence of 100 mM KCl for the mutated (**B**) and truncated (**C**) sequences. In (**B**) the height of the parallel peak at 262 nm was compared from the WT to the mutant sequences, where it is evident that the mutations involving the 3' region impacted G4 formation to a greater extent. (**C**) Truncated oligos were all less stable (indicated by lower T_M_s in the legend parentheses) with a decreased molar ellipticity. Moreover, the 3'-mid sequence has an apparent antiparallel shoulder, which is taken into account for modeling.

With seven contiguous runs of three or more continuous guanines, there are a number of potential G4s capable of forming. In order to determine which is the most likely principal structure, targeted G-to-T mutations of six of the seven runs of guanines was made (runs a, b, c, e, f, and g, Figure 4A), CD spectra were obtained, and molar ellipticity at the predominating parallel peak (262 nm) was compared (Figure 4B). Knocking out the 5'-end with the a23 sequence enhanced G4 formation, whereas the inability to form either the 5'-end or the 5'-mid sequences in the b2 sequence returned the molar ellipticity to WT baseline. All other mutations decreased G4 formation. The inability to form any mid sequences with the c2 and e2 sequences equally perturbed G4 formation, independent of the potential end runs available (3'-end and 5'-end, respectively). The most profound depression of molar ellipticity at 262 nm was found with the f23 sequence, which knocked out the 3'-mid and 3'-end formations; recovery of 3'-mid G4 formation with the g23 sequence increased molar ellipticity, but it was still markedly decreased as compared to WT and all other test sequences. Taken together, these data support the 3'-mid sequence to form one of the predominating G4s, but also indicate there is a complex environment facilitating multiple equilibrating formations. 

### 2.4. Resolution of Putative G4 Isoforms

Truncated 5'-end, 5'-mid, 3'-mid, and 3'-end sequences were examined in the presence of 100 mM KCl through CD spectra and thermal stability studies (Figure 4C). Each of these truncated sequences decreased the spectral signals for G4 formation, as compared to FL WT ADAM-15, but none were significantly affected more than the others. Within the 3'-mid spectra appeared an antiparallel shoulder, but the other structures supported all parallel species. Thermal changes were more evident. While all truncated sequences had a lower T_M_ than the FL WT (88 °C), intriguingly the lowest stability was noted in the 3'-mid sequence (72 °C), followed by the 3'-end (74 °C), the 5'-mid (76 °C) and by only a 3 °C decrease with the 5'-end sequence (85 °C).

**Figure 5:**
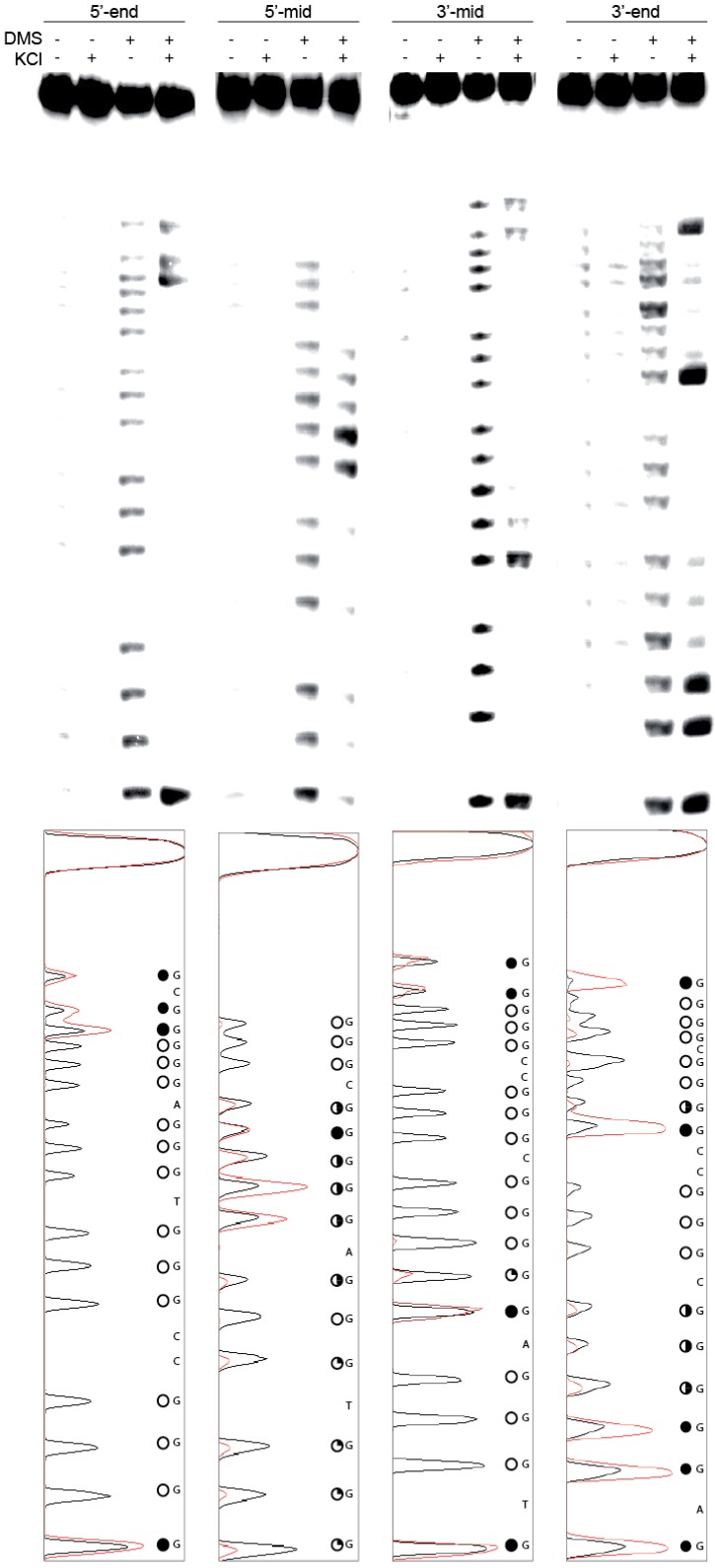
DMS footprinting of ADAM-15 truncated sequences 5'-end, 5'-mid, 3'-mid, 3'-end in the absence and presence of 140 mM KCl demonstrated the guanines involved in predominating G4 species. Histograms of each cleavage pattern are shown below in the absence (black line) and presence (red line) of KCl were used to determine the protection pattern indicated by the circles to the right of the figure: filled circle = hyper-cleaved, half-filled circle = partial protection indicative of protection within a subset of the equilibrating G4s, open circle = full protection indicative of incorporation in the majority of equilibrating G4 species.

**Figure 6:**
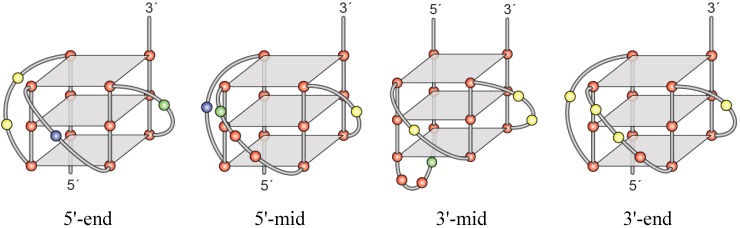
Models of the major G4 species from each truncated sequence (5'-end, 5'-mid, 3'-mid and 3'-end) were determined based on information from the CD and DMS footprinting data, including overall loop directionality (from CD) and majorly protected guanines (from DMS). Red = guanine, yellow = cytosine, blue = thymine, green = adenine.

DMS footprinting (Figure 5) firmly established the guanines utilized for G4 formation within each truncated sequence, and in concert with CD was used to model each of the four predominating isoforms (Figure 6). In the presence of 140 mM KCl, each of the four sequences demonstrated a guanine protection pattern of three continuous guanines within each of the four contiguous runs, supporting tri-tetrad stacks. The weakest protection patterns were found in the 5'-mid and 3'-end sequences, particularly within the fourth run of five guanines. 

A partial protection in a fourth guanine in this run with the 3'-mid sequence is suggestive of wobble, and supports more than one isoform forming even in the truncated runs, although a predominating pattern is evident. The particular loops for each isoform were found to be 2:1:1, 1:3:1, 3:1:2 and 1:3:1, for the 5'-end, 5'-mid, 3'-mid, and 3'-end sequences, respectively.

Truncation and mutation of the guanine-rich promoter region confirm that several equilibrating isoforms exist within the ADAM-15 promoter, including a strong contribution from the 3'-mid and 3'-end regions. Data obtained from chemical footprinting experiments were used to predict the major structures of the truncated regions, which will be useful in future molecular targeting and rational compound design. It is probable that more structures can form from the intact region containing all seven continuous guanine runs, not just from four contiguous runs as has been seen in other promoters, including kRAS and hTERT. More extensive examination of the individual mutations, and examination under supercoiled and other physiological conditions, are needed to fully elucidate all of the possible G4 isoforms, but the current works represent an initial description of the biological function and some likely major structures.

## 3. Experimental

### 3.1. Chemicals and Oligonucleotides

All oligonucleotides (Table 1) were synthesized by Eurofins Operon (Huntsville, AL, USA). All oligonucleotides were solvated in nuclease-free water upon arrival to a concentration of ~100 μM; final concentration was calculated by the nearest neighbor method using a Nano-Drop 2000 (Thermo Scientific, Waltham, MA, USA) to measure the absorbance at 260 nm (at 95 °C), and the extinction coefficient as calculated by the Integrated DNA Technologies server [24]. TMPyP4 was purchased from Calbiochem/Millipore (Billerica, MA, USA). All other chemicals, unless otherwise specified, were purchased through Fisher Scientific (Pittsburgh, PA, USA).

**Table 1:** Oligonucleotide sequences. G to T mutations are highlighted with bolded **T**’s.

Name	5'−3' Oligomer Sequence
FL	TTGGGGCCGGGTGGGAGGGGGCGGGCCGGGGCGGGGCC
5'-end	TTGGGGCCGGGTGGGAGGGGGCG
5'-mid	CCGGGTGGGAGGGGGCGGGCC
3'-mid	GTGGGAGGGGGCGGGCCGGGGCG
3'-end	GAGGGGGCGGGCCGGGGCGGGGCC
a23d3g23	TTG**TT**GCCGGGTGGGAGG**T**GGCGGGCCGGGGCG**TT**GCC
a23	TTG**TT**GCCGGGTGGGAGGGGGCGGGCCGGGGCGGGGCC
b2	TTGGGGCCG**T**GTGGGAGGGGGCGGGCCGGGGCGGGGCC
c2	TTGGGGCCGGGTG**T**GAGGGGGCGGGCCGGGGCGGGGCC
e2	TTGGGGCCGGGTGGGAGGGGGCG**T**GCCGGGGCGGGGCC
f23	TTGGGGCCGGGTGGGAGGGGGCGGGCCGG**T**GCGGGGCC
g23	TTGGGGCCGGGTGGGAGGGGGCGGGCCGGGGCG**TT**GCC

### 3.2. Circular Dichroism and Dimethyl Sulfate Footprinting

CD Spectra and thermal stability were measured on a J-810 spectropolarimeter (Jasco, Easton, MD, USA), as previously described [25]. DNA were diluted to 5 µM in a 50 mM Tris-HCl (pH 7.4) solution, with and without 100 mM KCl or NaCl. DMS footprinting was carried out as previously described [26]. Briefly, The 5'-end γ-^32^P-labeled DNA oligonucleotides were denatured by heating to 95 °C for 5 min and then slowly cooled at 4 °C in Tris-HCl buffer (50 mM, pH 7.4) with or without KCl (140 mM). Following the addition of 1 μg of calf thymus DNA, the DNA solutions were subjected to dimethyl sufate (0.5% DMS in 50% ethanol) for 18 min. Each reaction was quenched by adding 3.5 μL of DMSO. The reactions were subjected to preparative gel and each band of interest was excised and eluted in a buffer. After ethanol precipitation and treatment with piperidine, the cleaved products were separated on a 16% sequencing gel.

### 3.3. Plasmid Construction

Oligonucleotides (~40 bp each) containing the ADAM-15 WT or a23d3g23 sequences, plus the cut sites for insertion (6 bp each) into the pGL.3-Basic vector (Promega, Madison, WI, USA), namely 5'-KpnI and 3'-HindIII. Oligos were heated to 95 °C and cooled to their annealing temperatures where they were held for 5 min, and allowed to cool to room temperature. Annealed fragments were ligated into a KpnI/HindIII cut Basic vector backbone in a 5:1 ratio, transformed into DH5α competent *E. coli* (Thermo Fisher), and selected based on ampicillin resistance. Select clones were expanded, and plasmids were extracted with an endonuclease-free midi plasmid extraction kit (Qiagen, Valencia, CA, USA). Plasmids were confirmed with KpnI/HindIII digestion and extraction of the appropriately sized fragment, as well as with NheI digestion and the lack of a cut site.

### 3.4. Cell Culture, Transfection, Luciferase and PCR Assays

HEK-293 cells were maintained in a 37 °C, 5% CO2 incubator in exponential growth for the duration of the study in DMEM media (Life Technologies, Grand Island, NY, USA), supplemented with 10% FBS (Sigma Aldrich, St. Louis, MO, USA) and 1 × Penicillin/Streptomycin (Life Technologies). For transfection studies, cells were plated in 6-well plates at 2 × 10^5^ cells/well in 2 mL of media and allowed to attach overnight before co-transfection with the luciferase and control renilla vectors (Promega) in a 2:1 ratio using FuGene HD (Promega) in a 3:1 reagent:DNA ratio. Plasmids were allowed to transfect overnight, before TMPyP4 (100 µM) was added; after 48 h cells were lysed in passive lysis buffer. Luciferase expression was determined using a Dual Luciferase assay kit (Promega) on an automated luminometer (Berthold Technologies, Oak Ridge, TN, USA). Firefly luciferase expression was normalized to renilla luciferase expression. This ratio was then further normalized to WT controls, or samples in the presence of TMPyP4 were normalized to in the absence of compound, which was further normalized to compound effects noted with the empty vector (EV) plasmid. To examine whether any changes in luciferase expression were due to transcriptional or translational downregulation, PCR for firefly and renilla luciferase was performed following previously published methodology, with the modification of non-quantitative PCR with 30 cycles, and visualization as described below [26]. As above, 48 h post-treatment with TMPyP4, cells were lysed and RNA was harvested following the protocol or the RNeasy mini kit (Qiagen); 200 ng of cDNA was synthesized with iScript reverse transcriptase (Bio-Rad), and PCR products were visualized on a 1.5% agarose gel with 1× GelGreen. Expression was quantitated with ImageJ software (NIH, Bethesda, MD, USA), and firefly luciferase gene expression was normalized to renilla luciferase gene expression, and then further normalized as above. Experiments were repeated minimally in triplicate; statistical significance was determined by one-way ANOVA with a post-hoc Tukey test using GraphPad Prism software (GraphPad Software, La Jolla, CA, USA).

## 4. Conclusions

The core promoter of ADAM-15 encompassed within ~225 base pairs upstream of the TSS containing five Sp1 binding sites, 2 of which are overlapping with the G4-forming sequence [15]. This region is highly G/C-rich and putatively capable of forming higher order non-B-DNA structures *in vivo*. These G/C-rich regions preferentially cluster around the transcriptional start site throughout the genome, peaking within 50 bp up- and down-stream of the TSS [27,28], with a high prevalence in oncogenic promoters [29,30] including some representatives of the hallmarks of cancer [18]. Negative superhelicity induced by transcription can promote local unwinding of these G/C-rich regions of DNA, which allows for the formation of G4s, in a negative feedback-like system.

Formation of G4s in DNA has been recently shown to clearly form *in vivo*, where it modulates transcription and translation [31,32,33]. Their more unique, non-B-DNA, globular structure and potential to regulate the transcription of a host of oncogenes make G4s an attractive drug target for the development of anti-cancer agents. Due to varying loop lengths and composition, and number of stacked tetrads, drug design for G4 moieties is analogous to targeting a unique tertiary protein structure [34]. The targeting of G4 structures within oncogenic promoter regions has led to the development of two agents for clinical investigation. The first-in-class small molecule quarfloxin by Cylene Pharmaceuticals was halted at Phase II clinical development due to difficulties with delivery and excessive albumin binding, and Antisoma’s G-rich phosphodiester oligonucleotide AS1411 is in an ongoing clinical trial [27]. Such G4 targeting is a viable alternative approach to achieve the downregulation of numerous oncogenes, including ADAM-15.

Of the more than 20 members of the ADAM protein family, ADAMs-10, -15, and -17 are notable therapeutics targets for breast cancer [1]. Catalytic domain inhibitors for ADAMs-10 and -17 have been developed and are in clinical trials for breast cancer [13]. With the clear involvement of ADAM-15 in numerous aspects of tumorigenesis, invasion and metastasis, pharmaceutical targeting is attractive. Moreover, there is likely a greater therapeutic index with agents specific for ADAM-15, over ADAM-10 or -17, as it is not apparently involved in adult somatic cells and null mice demonstrated no overt phenotype [9]. In addition, the therapeutic intervention proposed currently has great potential for ADAM-15 specificity and thus a large therapeutic window.

ADAM-15 expression is increased in breast cancer, where its overexpression is coincidental with Her2/neu and a more aggressive and invasive phenotype [10,11,12]. As a disintegrin protein, it binds to a number of integrins, and in doing so alters cellular adhesion, migration, and metastasis [35,36,37,38]. Cell to cell adherence is mediated through cadherin proteins, and most family members have been demonstrated to be substrates of, or interactors with, ADAM-15, including N-, E-, and VE-cadherin [39,40,41]. The epithelial cell marker E-cadherin is cleaved into a smaller extracellular fragment, sE-cad, by ADAM-15. This soluble form can be found elevated in the serum of patients with metastatic cancers, including breast [42,43]. Of particular importance, the ADAM-15-mediated cleavage and release of sE-cad stimulates the Human epidermal growth factor receptor 2 (Her2/neu), a transmembrane protein overexpressed in 20%–25% of breast cancer patients. Oncogenic activation of Her2/neu leads to cellular proliferation and migration; higher levels of Her2/neu are associated with more invasive and aggressive breast cancer, and with lower rates of survival. The introduction of Herceptin, a Her2/neu extracellular domain-targeted therapeutic, to the marketplace has vastly improved outcome of patients with metastatic disease, as well as lowered recurrence rates and mortality in early-stage breast cancer [44]. However, there remains a vast majority of Her2/neu positive patients that do not respond to therapy [45]. For those patients who initially benefit, response is limited to a one-year duration [46,47].

ADAM-15 and HER2/neu were shown to be correspondingly upregulated in breast cancer in seven independent microarray studies [11]. This co-overexpression of both the HER2/neu receptor and ADAM-15 promotes a self-stimulating loop with the cleavage, release, and signaling of sE-cad. With enhanced autocrine signaling, these highly invasive and metastatic breast cancers carry a poor prognosis. A recent study demonstrated *in vivo* synergy between a HER2/neu-targeted agent and ADAM-15 knockdown with siRNA [14], highlighting the promise of targeting a single pathway at multiple points early in the signaling cascade. In combination with Herceptin, small molecules capable of targeted downregulation of ADAM-15 expression, such as those stabilizing the promoter G4, have the potential for synergism and a marked improvement on the lives of thousands of breast cancer patients a year.

## References

[1] Edwards D.R., Handsley M.M., Pennington C.J. (2008). The adam metalloproteinases. Mol. Asp. Med..

[2] Wolfsberg T.G., Straight P.D., Gerena R.L., Huovila A.P., Primakoff P., Myles D.G., White J.M. (1995). Adam, a widely distributed and developmentally regulated gene family encoding membrane proteins with a disintegrin and metalloprotease domain. Dev. Biol..

[3] Borrell-Pages M., Rojo F., Albanell J., Baselga J., Arribas J. (2003). Tace is required for the activation of the egfr by tgf-alpha in tumors. EMBO J..

[4] Kenny P.A., Bissell M.J. (2007). Targeting tace-dependent egfr ligand shedding in breast cancer. J. Clin. Investig..

[5] Moss M.L., Stoeck A., Yan W., Dempsey P.J. (2008). Adam10 as a target for anti-cancer therapy. Curr. Pharm. Biotechnol..

[6] Eto K., Huet C., Tarui T., Kupriyanov S., Liu H.Z., Puzon-McLaughlin W., Zhang X.P., Sheppard D., Engvall E., Takada Y. (2002). Functional classification of adams based on a conserved motif for binding to integrin alpha 9beta 1: Implications for sperm-egg binding and other cell interactions. J. Biol. Chem..

[7] Evans J.P. (2001). Fertilin beta and other adams as integrin ligands: Insights into cell adhesion and fertilization. Bioessays.

[8] Bohm B.B., Aigner T., Roy B., Brodie T.A., Blobel C.P., Burkhardt H. (2005). Homeostatic effects of the metalloproteinase disintegrin adam15 in degenerative cartilage remodeling. Arthritis Rheum..

[9] Horiuchi K., Weskamp G., Lum L., Hammes H.P., Cai H., Brodie T.A., Ludwig T., Chiusaroli R., Baron R., Preissner K.T. (2003). Potential role for adam15 in pathological neovascularization in mice. Mol. Cell. Biol..

[10] Kuefer R., Day K.C., Kleer C.G., Sabel M.S., Hofer M.D., Varambally S., Zorn C.S., Chinnaiyan A.M., Rubin M.A., Day M.L. (2006). Adam15 disintegrin is associated with aggressive prostate and breast cancer disease. Neoplasia.

[11] Najy A.J., Day K.C., Day M.L. (2008). The ectodomain shedding of e-cadherin by adam15 supports erbb receptor activation. J. Biol. Chem..

[12] Schutz A., Hartig W., Wobus M., Grosche J., Wittekind C., Aust G. (2005). Expression of adam15 in lung carcinomas. Virchows Arch..

[13] Lucas N., Day M.L. (2009). The role of the disintegrin metalloproteinase adam15 in prostate cancer progression. J. Cell. Biochem..

[14] Witters L., Scherle P., Friedman S., Fridman J., Caulder E., Newton R., Lipton A. (2008). Synergistic inhibition with a dual epidermal growth factor receptor/her-2/neu tyrosine kinase inhibitor and a disintegrin and metalloprotease inhibitor. Cancer Res..

[15] Khammas H., Bowen T., Williams J.D., Phillips A.O., Steadman R., Martin J. (2011). Characterisation of the human adam15 promoter. Nephron Exp. Nephrol..

[16] Verma A., Halder K., Halder R., Yadav V.K., Rawal P., Thakur R.K., Mohd F., Sharma A., Chowdhury S. (2008). Genome-wide computational and expression analyses reveal g-quadruplex DNA motifs as conserved *cis*-regulatory elements in human and related species. J. Med. Chem..

[17] Eddy J., Maizels N. (2009). Selection for the g4 DNA motif at the 5' end of human genes. Mol. Carcinog..

[18] Brooks T.A., Hurley L.H. (2009). The role of supercoiling in transcriptional control of myc and its importance in molecular therapeutics. Nat. Rev..

[19] Agarwala P., Pandey S., Mapa K., Maiti S. (2013). The g-quadruplex augments translation in the 5’ untranslated region of transforming growth factor beta2. Biochemistry.

[20] Bochman M.L., Paeschke K., Zakian V.A. (2012). DNA secondary structures: Stability and function of g-quadruplex structures. Nat. Rev. Genet..

[21] Marcel V., Tran P.L., Sagne C., Martel-Planche G., Vaslin L., Teulade-Fichou M.P., Hall J., Mergny J.L., Hainaut P., van Dyck E. (2011). G-quadruplex structures in tp53 intron 3: Role in alternative splicing and in production of p53 mrna isoforms. Carcinogenesis.

[22] Millevoi S., Moine H., Vagner S. (2012). G-quadruplexes in rna biology. Wiley Interdiscip. Rev. RNA.

[23] Qin Y., Hurley L.H. (2008). Structures, folding patterns, and functions of intramolecular DNA g-quadruplexes found in eukaryotic promoter regions. Biochimie.

[24] IDT Biophysics: UV Spectrum of DNA. http://biophysics.idtdna.com/UVSpectrum.html/.

[25] Qin Y., Fortin J.S., Tye D., Gleason-Guzman M., Brooks T.A., Hurley L.H. (2010). Molecular cloning of the human platelet-derived growth factor receptor β (pdgfr-β) promoter and drug targeting of the g-quadruplex-forming region to repress pdgfr-β expression. Biochemistry.

[26] Maekawa T., Sano Y., Shinagawa T., Rahman Z., Sakuma T., Nomura S., Licht J.D., Ishii S. (2008). Atf-2 controls transcription of maspin and gadd45 alpha genes independently from p53 to suppress mammary tumors. Oncogene.

[27] Balasubramanian S., Hurley L.H., Neidle S. (2011). Targeting g-quadruplexes in gene promoters: A novel anticancer strategy?. Nat. Rev. Drug Discov..

[28] Sjostrom J., Blomqvist C., von Boguslawski K., Bengtsson N.O., Mjaaland I., Malmstrom P., Ostenstadt B., Wist E., Valvere V., Takayama S. (2002). The predictive value of bcl-2, bax, bcl-xl, bag-1, fas, and fasl for chemotherapy response in advanced breast cancer. Clin. Cancer Res..

[29] Eddy J., Maizels N. (2006). Gene function correlates with potential for g4 DNA formation in the human genome. Nucleic Acids Res..

[30] MacLeod M.C. (1993). Identification of a DNA structural motif that includes the binding sites for sp1, p53 and ga-binding protein. Nucleic Acids Res..

[31] Biffi G., Tannahill D., McCafferty J., Balasubramanian S. (2013). Quantitative visualization of DNA g-quadruplex structures in human cells. Nat. Chem..

[32] Lam E.Y., Beraldi D., Tannahill D., Balasubramanian S. (2013). G-quadruplex structures are stable and detectable in human genomic DNA. Nat. Commun..

[33] Muller S., Kumari S., Rodriguez R., Balasubramanian S. (2010). Small-molecule-mediated g-quadruplex isolation from human cells. Nat. Chem..

[34] Brooks T.A., Kendrick S., Hurley L. (2010). Making sense of g-quadruplex and i-motif functions in oncogene promoters. FEBS J..

[35] Eto K., Puzon-McLaughlin W., Sheppard D., Sehara-Fujisawa A., Zhang X.P., Takada Y. (2000). Rgd-independent binding of integrin alpha9beta1 to the adam-12 and -15 disintegrin domains mediates cell-cell interaction. J. Biol. Chem..

[36] Nath D., Slocombe P.M., Stephens P.E., Warn A., Hutchinson G.R., Yamada K.M., Docherty A.J., Murphy G. (1999). Interaction of metargidin (adam-15) with alphavbeta3 and alpha5beta1 integrins on different haemopoietic cells. J. Cell Sci..

[37] Trochon-Joseph V., Martel-Renoir D., Mir L.M., Thomaidis A., Opolon P., Connault E., Li H., Grenet C., Fauvel-Lafeve F., Soria J. (2004). Evidence of antiangiogenic and antimetastatic activities of the recombinant disintegrin domain of metargidin. Cancer Res..

[38] Zhang X.P., Kamata T., Yokoyama K., Puzon-McLaughlin W., Takada Y. (1998). Specific interaction of the recombinant disintegrin-like domain of mdc-15 (metargidin, adam-15) with integrin alphavbeta3. J. Biol. Chem..

[39] Ham C., Levkau B., Raines E.W., Herren B. (2002). Adam15 is an adherens junction molecule whose surface expression can be driven by ve-cadherin. Exp. Cell Res..

[40] Najy A.J., Day K.C., Day M.L. (2008). Adam15 supports prostate cancer metastasis by modulating tumor cell-endothelial cell interaction. Cancer Res..

[41] Nieman M.T., Prudoff R.S., Johnson K.R., Wheelock M.J. (1999). N-cadherin promotes motility in human breast cancer cells regardless of their e-cadherin expression. J. Cell Biol..

[42] Gofuku J., Shiozaki H., Doki Y., Inoue M., Hirao M., Fukuchi N., Monden M. (1998). Characterization of soluble e-cadherin as a disease marker in gastric cancer patients. Br. J. Cancer.

[43] Kuefer R., Hofer M.D., Gschwend J.E., Pienta K.J., Sanda M.G., Chinnaiyan A.M., Rubin M.A., Day M.L. (2003). The role of an 80 kda fragment of e-cadherin in the metastatic progression of prostate cancer. Clin. Cancer Res..

[44] Bedard P.L., Piccart-Gebhart M.J. (2008). Current paradigms for the use of her2-targeted therapy in early-stage breast cancer. Clin. Breast Cancer.

[45] Vogel C.L., Cobleigh M.A., Tripathy D., Gutheil J.C., Harris L.N., Fehrenbacher L., Slamon D.J., Murphy M., Novotny W.F., Burchmore M. (2002). Efficacy and safety of trastuzumab as a single agent in first-line treatment of her2-overexpressing metastatic breast cancer. J. Clin. Oncol..

[46] Dieras V., Vincent-Salomon A., Degeorges A., Beuzeboc P., Mignot L., de Cremoux P. (2007). trastuzumab (herceptin) and breast cancer: Mechanisms of resistance. Bull. Cancer.

[47] Mariani G., Fasolo A., de Benedictis E., Gianni L. (2009). Trastuzumab as adjuvant systemic therapy for her2-positive breast cancer. Nat. Clin. Pract..

